# Activation-Induced TIM-4 Expression Identifies Differential Responsiveness of Intestinal CD103+ CD11b+ Dendritic Cells to a Mucosal Adjuvant

**DOI:** 10.1371/journal.pone.0158775

**Published:** 2016-07-05

**Authors:** Kerry L. Hilligan, Lisa M. Connor, Alfonso J. Schmidt, Franca Ronchese

**Affiliations:** 1 Malaghan Institute of Medical Research, Wellington, New Zealand; 2 School of Biological Sciences, Victoria University of Wellington, Wellington, New Zealand; Oklahoma Medical Research Foundation, UNITED STATES

## Abstract

Macrophage and dendritic cell (DC) populations residing in the intestinal lamina propria (LP) are highly heterogeneous and have disparate yet collaborative roles in the promotion of adaptive immune responses towards intestinal antigen. Under steady-state conditions, macrophages are efficient at acquiring antigen but are non-migratory. In comparison, intestinal DC are inefficient at antigen uptake but migrate to the mesenteric lymph nodes (mLN) where they present antigen to T cells. Whether such distinction in the roles of DC and macrophages in the uptake and transport of antigen is maintained under immunostimulatory conditions is less clear. Here we show that the scavenger and phosphatidylserine receptor T cell Immunoglobulin and Mucin (TIM)-4 is expressed by the majority of LP macrophages at steady-state, whereas DC are TIM-4 negative. Oral treatment with the mucosal adjuvant cholera toxin (CT) induces expression of TIM-4 on a proportion of CD103+ CD11b+ DC in the LP. TIM-4+ DC selectively express high levels of co-stimulatory molecules after CT treatment and are detected in the mLN a short time after appearing in the LP. Importantly, intestinal macrophages and DC expressing TIM-4 are more efficient than their TIM-4 negative counterparts at taking up apoptotic cells and soluble antigen *ex vivo*. Taken together, our results show that CT induces phenotypic changes to migratory intestinal DC that may impact their ability to take up local antigens and in turn promote the priming of mucosal immunity.

## Introduction

The intestine is a complex immune organ equipped with highly specialised and unique populations of antigen presenting cells (APC), including dendritic cells (DC) and macrophages in the lamina propria (LP). These APC are required to maintain tolerance to food proteins and commensal microbes but retain the capacity to recognise and eliminate pathogens. Extensive study has focussed on characterising intestinal APC at steady-state and understanding the roles of individual APC subsets in promoting peripheral tolerance [1–4]. However, the phenotypic and functional characteristics of DC and macrophages under immunostimulatory conditions are less clear.

The presence of pathogens or inflammatory agents in the intestine is associated with the initiation of active adaptive immunity rather than the induction of peripheral tolerance towards a given antigen [4–9]. DC are effective APC that interpret signals from microbial stimuli through pattern recognition receptors leading to activation, licensing and increased expression of co-stimulatory molecules (CD80/CD86) necessary for effector T cell proliferation and differentiation [10, 11]. DC then migrate from the LP to the mesenteric lymph nodes (mLN) to prime naïve T cells against antigen encountered in the intestine. Interestingly, experimental data suggests that antigen uptake and migration to the LN are carried out by distinct APC subsets. DC efficiently migrate to the mLN [12–17], however, antigen uptake in this population has been very difficult to detect. CX3CR1+ macrophages are highly efficient at acquiring intestinal antigen [18], but they are not found in the mLN and are inefficient at priming naïve T cells *in vitro* [12, 13]. Albeit CX3CR1 cells have been detected in the afferent lymph [19, 20], the identification of this population as macrophages can be debated as a recent study identified a novel LP DC population expressing CX3CR1 but lacking CD64 or CD103 expression [21].

The disparity in the roles of intestinal macrophages and DC in the uptake and presentation of antigen has resulted in much debate over the potential mechanisms by which DC acquire antigen for transport to the mLN and several possibilities exist. In the presence of microbial stimuli, DC may adapt to become more efficient at acquiring antigen. For example, *Salmonella typhimurium* in the lumen of the intestine was shown to promote localisation of LP CD103+ DC to the epithelial barrier and the extension of dendrites into the luminal space [22]. Conversely, at steady-state, DC appear to obtain antigen through interactions with neighbouring cells more proficient at antigen uptake, such as goblet cells and CX3CR1+ macrophages [18, 23]. Given these findings, apoptotic epithelial cells may play an important role in antigen transfer to DC as epithelial cells have been detected taking up large quantities of orally delivered antigen [18] and LP derived DC have been shown to present apoptotic-associated antigens to T cells in the mLN [15]. Overall, it is likely that multiple pathways of antigen uptake are involved and further studies are required to resolve this question.

T cell immunoglobulin and mucin (TIM)-4 is a protein expressed by APC [24–27] known to interact with phosphatidylserine (PtdSer) [28] and TIM-1 [25]. Functional characteristics attributed to TIM-4 include apoptotic cell recognition and uptake [28–31], transfer of material between cells [32] and T cell co-stimulation [24–27]. Furthermore, DC exposed to microbial products *in vitro* have been reported to express increased levels of TIM-4 [26, 27, 33]. Thus, TIM-4 is an interesting candidate protein to study in the context of antigen uptake, transfer and presentation by intestinal APC populations under immunostimulatory conditions.

In this study, we modelled an immunostimulatory environment within the intestine by orally administering the mucosal adjuvant cholera toxin (CT) to gain an understanding of the APC and processes involved in promoting intestinal effector responses. We demonstrate that a proportion of LP CD103+ CD11b+ DC up-regulate TIM-4 and co-stimulatory molecules in response to CT, and migrate to the mLN. Expression of TIM-4 is associated with an enhanced capacity of APC to take up apoptotic material and soluble antigen *ex vivo*. We propose that CD103+ CD11b+ TIM-4+ DC represent a population of mature DC that are actively responding to CT and alter their functional capacities to better acquire antigen. Therefore, CD103+ CD11b+ TIM-4+ DC are likely contributing to the development of immunity in the presence of CT.

## Materials and Methods

### Mice

Specific pathogen-free C57BL/6 mice were obtained from Jackson Laboratories (Bar Harbor, ME) and bred and maintained at the Malaghan Institute of Medical Research. Female mice of 6–8 weeks of age were used for all experiments. All experimental protocols were approved by the Victoria University Animal Ethics Committee and performed according to institutional guidelines.

### *In vivo* treatments

To induce immune stimulation, mice received 10μg cholera toxin (CT, Sigma-Aldrich) in 250μL bicarbonate buffer (pH 9.6) by oral gavage. To assess DC migration, 100μL 10mM 5- and 6-carboxyfluorescein diacetate succinimidyl ester (CFDA-SE; Molecular Probes, Invitrogen) was administered in DMSO by oral gavage 20 minutes after CT treatment.

### Lamina propria cell isolation

Jejunal sections were isolated and flushed with Hank’s Balanced Salt Solution (HBSS, Invitrogen) and the Peyer’s patches excised. Sections were opened longitudinally and incubated twice in HBSS containing 10% FCS (Invitrogen), 2mM EDTA and 25mM Hepes buffer (both from Sigma-Aldrich) in a 37°C Innova 4200 Incubator Shaker (Edison, NJ) at 150rpm for 15 minutes. Tissue was then minced with scissors and digested in IMDM (Invitrogen) containing 57.5μg/mL Liberase TL (Roche) for 45 minutes in a shaking incubator. Cells were then washed and passed through a 40μm cell strainer.

### Apoptotic cell uptake assay

A single cell suspension was prepared from the thymus of juvenile mice. Thymocytes were labelled with eFluor670 dye (eBioscience, San Diego, CA) and apoptosis was induced by incubating in IMDM containing 5μM dexamethasone (Sigma-Aldrich) for 4 hours at room temperature. Apoptotic cells were identified by Annexin-V and PI staining (both from BD Pharmingen, San Jose, CA). Small intestinal APC were isolated from the LP of untreated or CT-treated mice and enriched by positive magnetic selection using anti-CD11c microbeads (Miltenyi Biotec, Germany). APC were incubated at 37°C with apoptotic thymocytes for 1 hour and stained for cell surface proteins and analysed for eFluor670 fluorescence by flow cytometry.

### *In vitro* antigen uptake assay

CD11c+ cells from the small intestine of untreated and CT-treated mice were isolated by positive magnetic selection and incubated with 25μg/mL OVA-AF488 (Molecular Probes) at 4°C or 37°C for 45 minutes. Cells were then stained with fluorescent antibodies against cell surface molecules and assessed for uptake of OVA-AF488 by flow cytometry.

### Flow cytometry

Multicolour flow cytometry was performed on single cell suspensions prepared from the LP and mesenteric LN (mLN) of untreated and CT-treated mice. To prevent non-specific binding of monoclonal antibodies, cells were incubated with anti-mouse CD16/32 (clone 2.4G2, affinity purified from hybridoma culture supernatant) prior to labelling cocktails of fluorescent antibodies made up in PBS containing 2mM EDTA, 0.01% sodium azide and 2% FCS. Anti-CD11c (HL3), anti-CD86 (GL-1) and anti-CD103 (M290) were from BD Pharmingen (San Jose, CA). Anti-CD11b (M1/70), anti-CD24 (M1/69), anti-CD26 (H194-112), anti-CD64 (X54-5/7.1), anti-CD88 (C5aR, clone 20/70), anti-CD273 (PD-L2, clone TY25) and anti-TIM-4 (RMT4-54) were from BioLegend (San Diego, CA). Anti-CD274 (PD-L1, clone MIH5), anti-F4/80 (BM-8) were from eBioscience (San Diego, CA) and anti-MHCII (3JP) were prepared in-house. To visualize biotinylated antibodies, cells were washed following labelling with primary monoclonal antibodies and incubated with streptavidin conjugated to PE-Texas Red or PECF594 (BD Pharmingen, San Jose, CA).

For assessment of CX3CR1 expression, cells were incubated with CX3CR1-Fc fusion protein neurotactin (kindly provided by Prof. Steffen Jung, Rehovot, Israel), followed by goat-anti-human IgG1 conjugated to APC (Jackson ImmunoResearch Laboratories, PA) prior to staining for other cell surface molecules.

For assessment of aldehyde dehydrogenase activity, cells were stained using an ALDEFLUOR™ kit (Stem Cell Technologies). Positive cells were determined by comparison with aldehyde dehydrogenase negative control, which was generated by the addition of aldehyde dehydrogenase inhibitor diethylaminobenzaldehyde (DEAB) to control samples prior to staining with ALDEFLUOR™.

Compensation was set in each experiment using BD CompBeads^™^ (BD Pharmingen) and dead cells were identified and excluded from analysis using DAPI labelling (Molecular Probes, Invitrogen). All samples were collected on a LSRFortessa SORP^™^ flow cytometer (Becton Dickinson, San Jose, CA) and analysed using FlowJo software (version 9.6.2., Treestar Inc).

### Morphology studies

APC were isolated from the LP of CT treated mice and DAPI- CD11c+ MHCII+ CD11b+ cells were sorted into CD103+ TIM-4−, CD103+ TIM-4+ and CD103- TIM-4+ populations using a FACSVantage DiVa^™^ (Becton Dickinson, San Jose, CA). Each sorted population was plated onto a slide using a Shandon CytoSpin 4 centrifuge (Thermo Scientific, USA) and stained with a Diff-Quik kit (Siemens, Germany). Bright field images were acquired using a 100x objective lens on a compound BX51 microscope and images were captured with a DP70 digital camera (both from Olympus, Center Valley, PA).

### Statistical analysis

Statistical analyses were performed using Prism 5.0 GraphPad Software. Mean ± SEM is shown on all graphs. Data were analyzed using One-way ANOVA with Bonferroni post-test or Student’s unpaired t-test; *p* values lower than 0.05 were considered statistically significant.

## Results

### TIM-4 is expressed by macrophages but not DC in the steady-state SI LP

**Fig 1:**
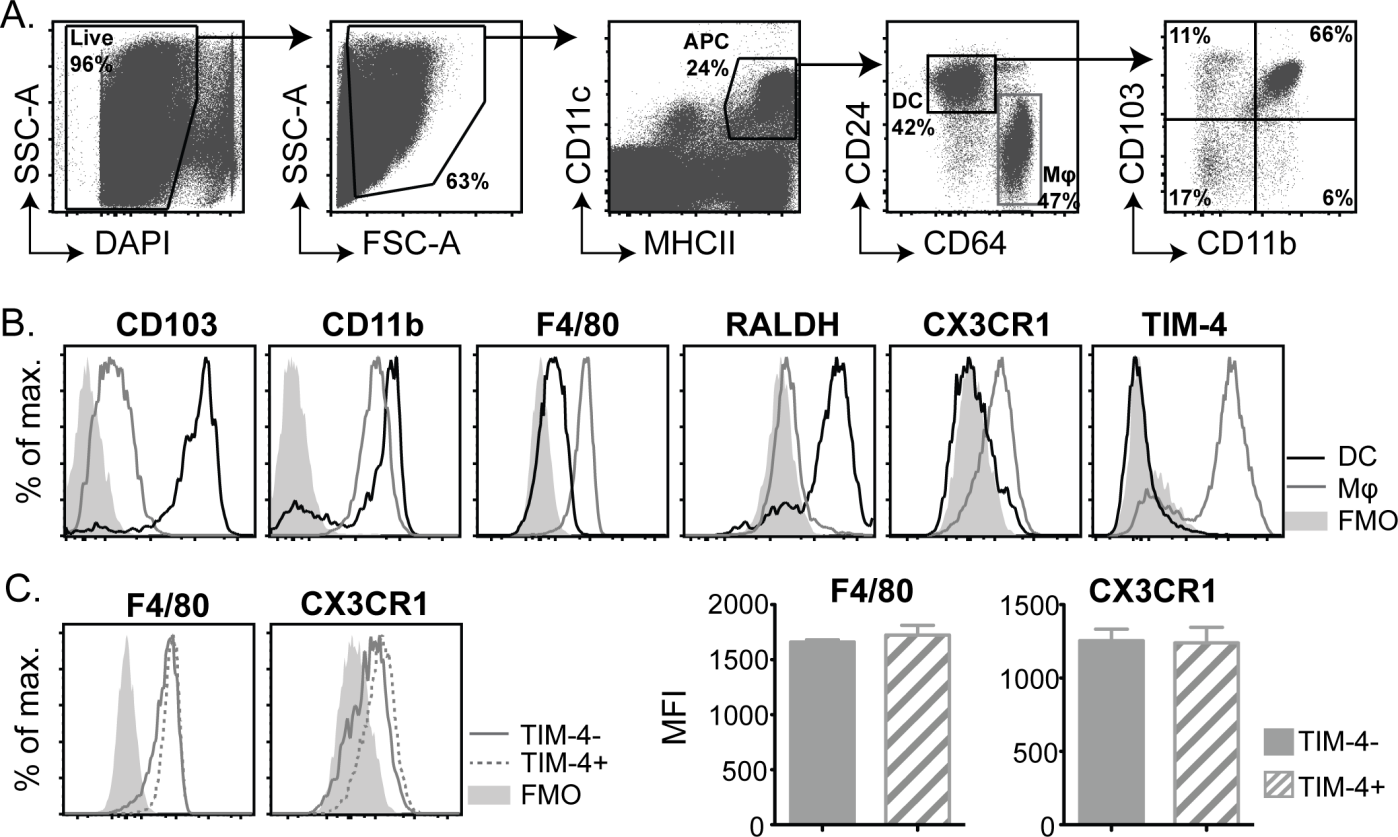
Macrophages, but not DC, from the SI LP express TIM-4 at steady-state. APC were isolated from the SI of untreated mice and analysed for expression of cell surface molecules by flow cytometry. (A) Dot plots show the gating strategy employed to identify distinct SI DC and macrophage (Mφ) populations. (B) Histograms compare expression profiles of cell surface molecules and RALDH between DC and macrophage populations identified as in (A). (C) Intestinal macrophages were divided into TIM-4− and TIM-4+ populations and compared for expression of F4/80 and CX3CR1. Bar graphs show average median fluorescent intensity (MFI)±SEM for 3 individual mice. Data are from 1 of 3 independent experiments, each with 3 mice, that gave similar results.

To gain a better understanding of the composition of the APC population present in the steady-state small intestine (SI), we analysed CD11c+ MHCII+ cells isolated from the jejunal lamina propria (LP) by flow cytometry. In line with previous reports, DC and macrophages could be clearly distinguished based on the cell surface expression of CD24 and CD64 respectively [34] (Fig 1A). Interestingly, expression of CD24 and CD64 directly correlated with the cell surface expression of CD26 and CD88 [35] (S1A and S1B Fig).

Consistent with their classification as DC, CD24+ cells had high activity of the DC-associated enzyme RALDH and lacked expression of the macrophage-associated markers F4/80 and CX3CR1 (Fig 1B and S1C Fig). The majority of CD24+ cells co-expressed CD103 and CD11b but other DC subsets namely: CD103+ CD11b−, CD103- CD11b+ and CD103− CD11b−, were also detected at low frequencies (Fig 1A). In contrast, CD64+ cells expressed F4/80 and CX3CR1 and lacked expression of CD103 and RALDH, confirming their identification as macrophages (Fig 1B and S1C Fig).

We also examined expression of the PtdSer receptor TIM-4, which is expressed by DC and macrophages in other tissues [28, 29, 36]. Expression of TIM-4 clearly defined two subpopulations of SI macrophages, whereas there was no TIM-4 detected on steady-state SI DC (Fig 1B). TIM-4+ macrophages accounted for approximately 75% of the total macrophage population and had comparable expression of F4/80 and CX3CR1 to TIM-4− macrophages (Fig 1C). Overall, this data highlights the complexity and heterogeneity of SI APC with four distinct DC and two distinct macrophage subsets clearly identified in the steady-state LP.

### CT promotes the expression of cell surface TIM-4 expression by CD103+ CD11b+ DC

**Fig 2:**
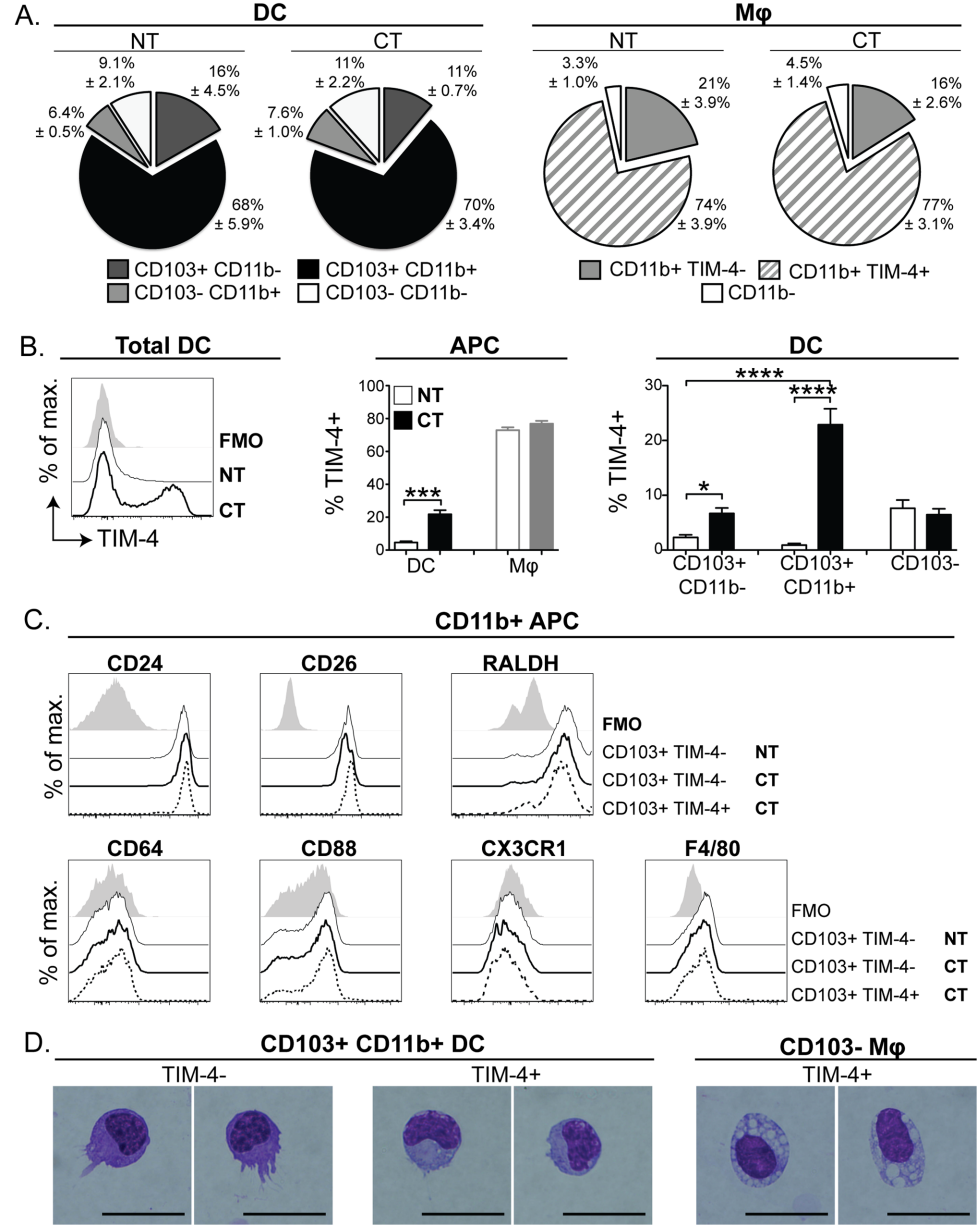
CD103+ CD11b+ DC in the SI LP express TIM-4 following oral delivery of CT. SI APC were isolated from mice treated with CT 17 hours prior to analysis and untreated control mice (NT), and gated into DC and macrophage populations as in Fig 1A. (A) Pie charts show the mean frequency of individual DC and macrophage subsets. Mean±SEM was calculated using data compiled from 5 independent experiments, each with 3 mice per group and is shown alongside charts. (B) Total DC were assessed for TIM-4 expression by flow cytometry. Bar graphs show average frequency of TIM-4+ cells in each DC subset ±SEM for 3 individual mice. Statistical significance was determined by one-way ANOVA with Bonferroni post-test. * p <0.05, *** 0.0001<p<0.001, **** p<0.0001. (C) CD103+ CD11b+ TIM-4+ DC (dashed black lines) were compared to CD103+ CD11b+ TIM-4− DC from untreated (NT, black lines) and CT treated (bolded black lines) animals for expression of cell surface molecules and RALDH activity by flow cytometry. Data are from 1 of 3 independent experiments, each with 3 mice per group, that gave similar results. (D) Cytospin images are of Diff-Quik stained SI APC isolated from CT treated mice and sorted into CD103+ CD11b+ TIM-4− DC, CD103+ CD11b+ TIM-4+ DC and TIM-4+ macrophage populations by FACS. Scale bar = 20μm.

We were interested in determining whether the phenotype and subset composition of SI APC was altered following the introduction of a mucosal adjuvant. CT is known to promote the development of effector T cells in the mLN and overcome tolerance induction towards co-administered antigen [1, 7, 37]. We isolated SI LP cells 17 hours following CT treatment and analysed them for expression of cell surface markers by flow cytometry. CT treatment did not significantly affect the composition of the SI APC population with the frequency (Fig 2A) and number (S2A and S2B Fig) of individual DC and macrophage subsets remaining similar to steady-state. Thus, this finding suggests that there was no selective expansion of a particular APC subset during the early response to a mucosal adjuvant.

Comparison of APC phenotype in the steady-state and after CT treatment revealed an abundant population of SI CD103+ CD11b+ DC expressing the PtdSer receptor TIM-4, which was not present in steady-state SI LP (Fig 2B). Increased expression of TIM-4 was also observed on the CD103+ CD11b− subset; however, this change was less profound than that observed in the CD103+ CD11b+ population. CD103+ CD11b+ TIM-4+ cells also expressed CD24, CD26 and stable levels of RALDH, and were negative for the macrophage-associated markers CD64, CD88, CX3CR1 and F4/80 (Fig 2C) confirming their putative identification as DC. Diff-Quik staining of sorted SI LP cells revealed that CD103+ TIM-4+ cells were morphologically distinct from CD103− TIM-4+ cells, which have a vacuolar cytoplasm resembling that of macrophages. In addition, CD103+ TIM-4+ cells also lacked prominent dendrite protrusions, which are typical of DC and were present on CD103+ TIM-4− cells (Fig 2D). Together these data suggest that a proportion of the predominant CD103+ CD11b+ DC population express TIM-4 in response to CT.

### CD103+ CD11b+ DC up-regulate co-stimulatory molecules in addition to TIM-4

**Fig 3:**
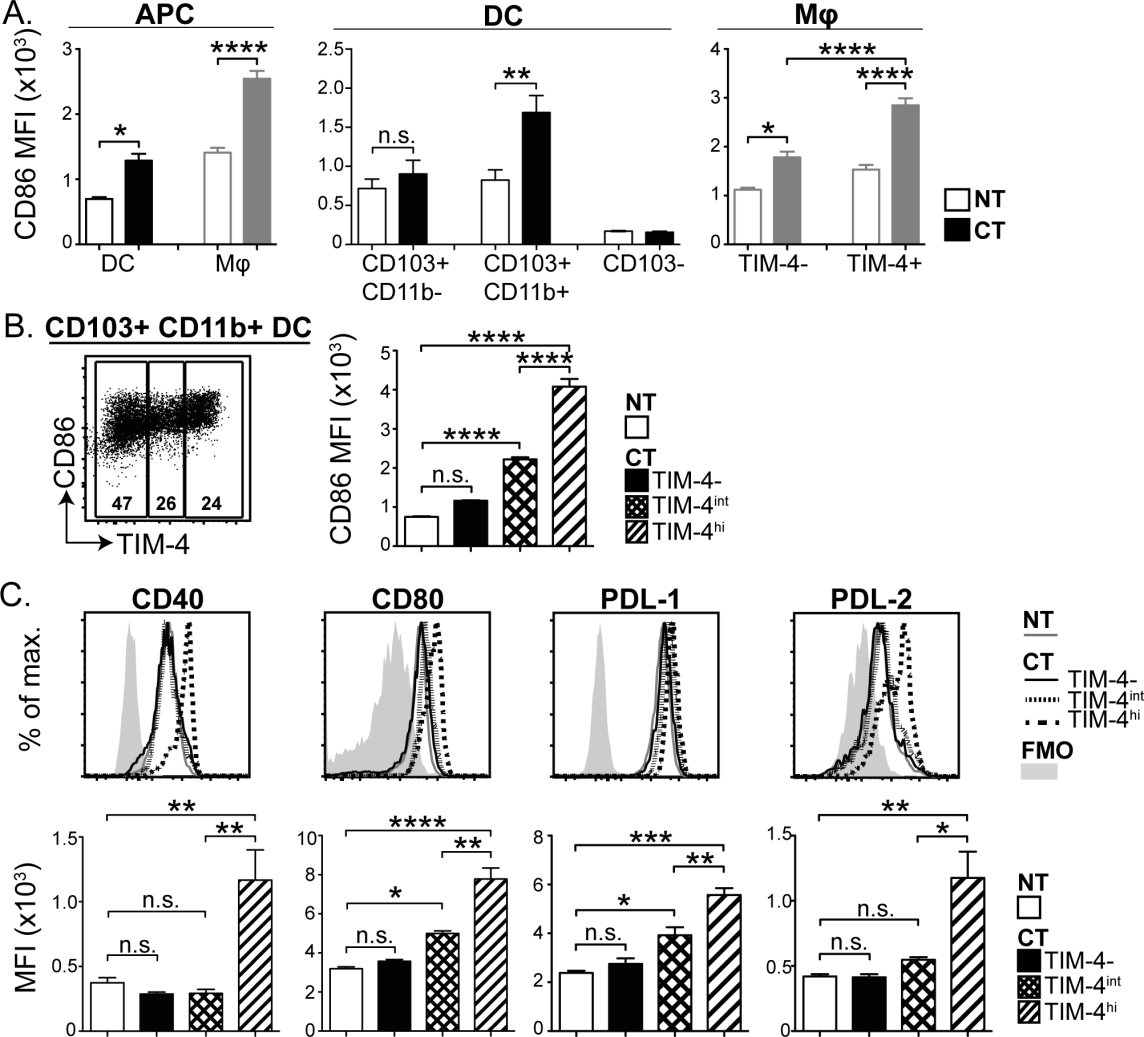
CD103+ CD11b+ TIM-4+ DC in the SI LP express increased levels of co-stimulatory molecules compared to TIM-4− DC. (A) SI DC and macrophage subsets from untreated (NT) and CT treated mice were identified using flow cytometry and compared for expression of CD86. (B) CD103+ CD11b+ DC from CT treated mice were divided into TIM-4−, TIM-4^int^ and TIM-4^hi^ subsets and compared to untreated CD103+ CD11b+ TIM-4− DC for expression of CD86. (C) As in B, except that CD40, CD80, PDL-1 and PDL-2 expression was compared. Bar graphs show average median fluorescence intensity (MFI)±SEM for 3 mice. Statistical significance was determined by one-way ANOVA with Bonferroni post-test. n.s. p >0.05, * p <0.05, ** 0.001<p<0.05, *** 0.0001<p<0.001, **** p<0.0001.

To further assess the response to CT, we examined co-stimulatory molecule expression on APC subsets following CT treatment. Both DC and macrophages significantly up-regulated CD86 in response to CT. Analysis of individual DC subsets showed that CD86 was selectively up-regulated among the predominant CD103+ CD11b+ population. Both TIM-4− and TIM-4+ macrophages significantly up-regulated CD86; however, the increase was more pronounced within the TIM-4+ subset (Fig 3A).

Given the specific activation and TIM-4 up-regulation among CD103+ CD11b+ DC, we were interested in determining whether there was a correlation in the expression of TIM-4 and the activation status of the DC. To do this, we subdivided the CD103+ CD11b+ population into TIM-4−, TIM-4^int^ and TIM-4^hi^ subsets and assessed each individually for expression of co-stimulatory molecules. TIM-4^hi^ DC had substantially higher expression of CD86 compared to TIM-4^int^ and TIM-4− DC. In fact, expression of CD86 on TIM-4− DC was similar to that of CD103+ CD11b+ DC from untreated mice (Fig 3B). Expression of other co-stimulatory molecules, CD40, CD80, PDL-1 and PDL-2 showed a similar trend, with CD103+ CD11b+ TIM-4^hi^ DC expressing significantly higher levels of these markers compared to CD103+ CD11b+ DC from control mice (Fig 3C). CD103+ CD11b− and CD103− DC did not up-regulate CD40 or CD80 in response to CT (S3A and S3B Fig). Of note, TIM-4^int^ DC showed higher expression of some co-stimulatory molecules but not others, suggesting distinct expression kinetics among different co-stimulatory molecules (Fig 3B and 3C).

Next, we wanted to address whether the CD103+ CD11b+ TIM-4+ DC were a population of LP resident DC responding to CT, or a DC population newly recruited to the SI. To this purpose, we isolated DC from the SI of untreated mice and cultured them overnight in the presence of CT or the TLR4 ligand LPS. Regardless of *in vitro* treatment, all cultured CD103+ CD11b+ DC up-regulated CD86 compared to the pre-culture control. In line with our *in vivo* observations, a proportion CD103+ CD11b+ DC also up-regulated TIM-4 compared to the pre-culture control (S3C Fig) supporting our conclusion that TIM-4 is associated with the activation of LP DC. Further to this, we tracked the expression of CD86 and TIM-4 on CD103+ CD11b+ DC over time after CT treatment *in vivo*. There was a clear shift of cells from a CD86- TIM-4- phenotype at baseline to a CD86+ TIM-4+ phenotype by 17 hours post-treatment (S3D Fig). Together, this data supports the conclusion that LP resident CD103+ CD11b+ DC selectively up-regulate TIM-4 concurrently with co-stimulatory molecules.

### CD103+ CD11b+ TIM-4+ DC present in the mLN are a population of migratory DC

**Fig 4:**
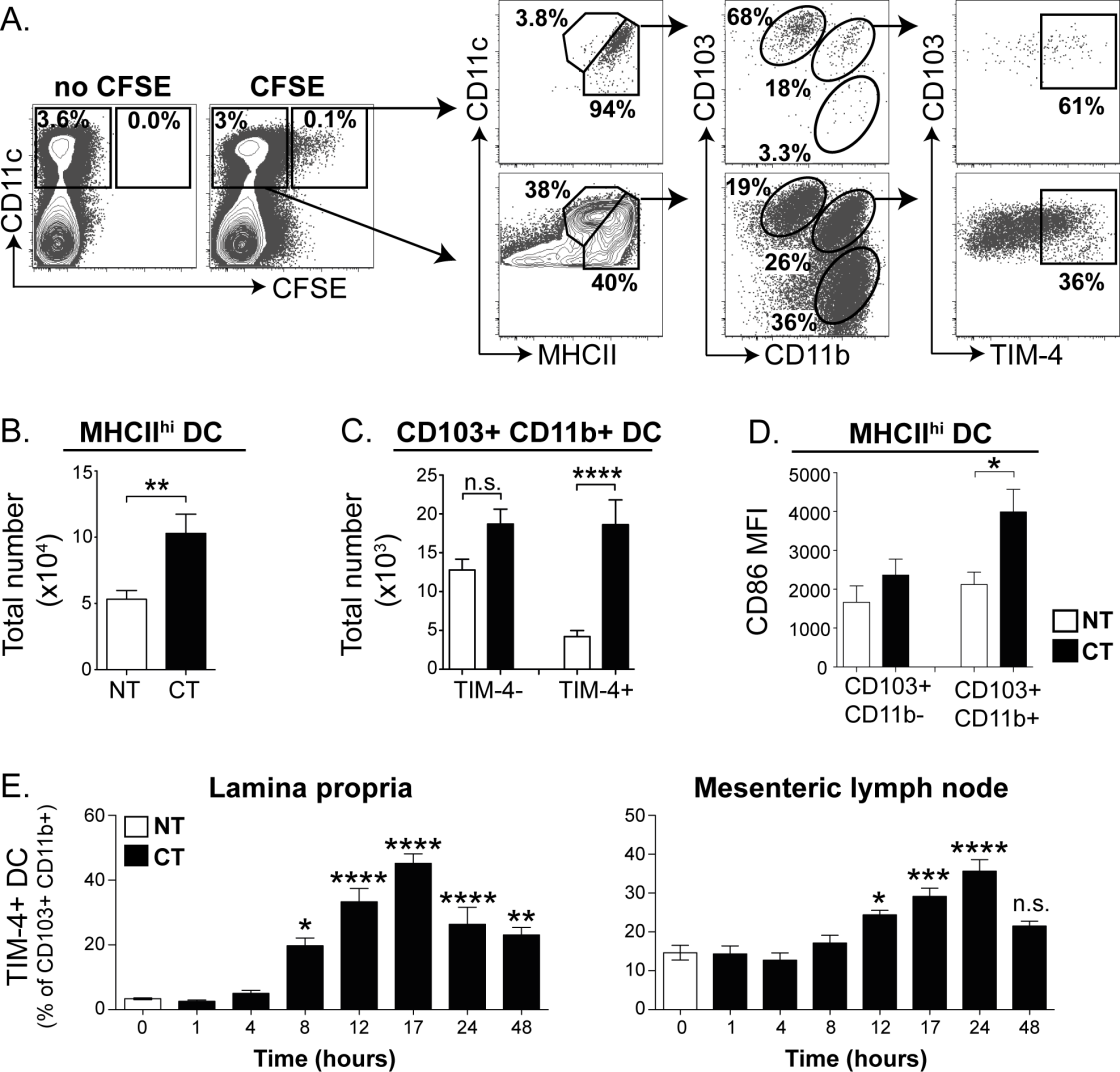
CD103+ CD11b+ TIM-4+ DC are enriched in the mLN of CT treated mice suggesting migration from the LP. (A) Cells isolated from the mLN were assessed for expression of CFSE 17 hours after oral administration of CT and CFDA-SE. (B) mLN DC subset frequencies from untreated (NT) and CT-treated mice were identified by flow cytometry and used to calculate cell numbers. Bar graph shows average total number of CD11c+ MHCII^hi^ DC in the mLN ±SEM for 9 mice from 3 independent experiments with 3 mice per group. Statistical significance was determined by Student’s unpaired t-test. *** 0.0001<p<0.001 (C) As in B, except numbers of CD103+ CD11b+ TIM-4− and CD103+ CD11b+ TIM-4+ DC are shown. (D) CD103+ CD11b− and CD103+ CD11b+ mLN DC were compared for expression of CD86 by flow cytometry. Bar graphs show average median fluorescence intensity (MFI)±SEM for 3 mice. (E) DC were isolated from the SI LP and mLN of untreated mice (NT) and mice administered CT at different time points and analysed for TIM-4 expression by flow cytometry. Bar graphs show average frequency of TIM-4+ DC among total CD103+ CD11b+ cells ±SEM pooled from 3 independent experiments with 3 mice per group. Statistical significance was determined by one-way ANOVA with Bonferroni post-test. n.s. p >0.05, * p <0.05, ** 0.001<p<0.05, *** 0.0001<p<0.001, **** p<0.0001 as compared to DC from untreated mice.

In addition to up-regulating co-stimulatory molecules, SI DC must migrate to the mLN in order to present luminal antigen to T cells and promote effector T cell responses. Therefore, it was of interest to compare the composition of mLN migratory DC subsets between untreated and CT-treated mice. However, a method of identifying migratory DC in the mLN has not been corroborated. Migratory DC have previously been distinguished from LN resident DC by their high expression of MHCII in the mLN as well as the mediastinal and skin dLN [6, 21, 38]. To verify that this approach could be used reliably in the identification of migratory DC in the mLN, we orally administered the CFSE precursor CFDA-SE following CT treatment and assessed mLN cells for CFSE fluorescence 17 hours later. We found that more than 90% of CFSE+ cells in the mLN were MHCII^hi^ DC. CFSE+ MHCII^hi^ cells consisted of CD103+ CD11b- and CD103+ CD11b+ DC subsets, a proportion of which expressed TIM-4 (Fig 4A). This data suggests that MHCII^hi^ DC, including the CD103+ CD11b+ TIM-4+ subset, are a migrant DC population from the LP rather than a LN resident population.

### CD103+ CD11b+ TIM-4+ DC migrate to the mLN following CT administration

Having established a gating strategy for identifying migratory DC subsets in the mLN, we sought to assess the numbers and phenotype of migratory DC in the mLN following CT treatment. The overall number of CD11c+ MHCII^hi^ cells was increased following CT treatment (Fig 4B). Importantly, this change was due to a selective increase in the number of CD103+ TIM-4+ cells in the mLN (Fig 4C). In addition, among migratory MHCII^hi^ mLN DC, only CD103+ CD11b+ DC and not CD103+ CD11b− DC showed increased expression of CD86 following CT (Fig 4D), further suggesting that the CD103+ CD11b+ DC population is preferentially responding to CT.

To gain a better understanding of the kinetics of the presence of activated CD103+ CD11b+ DC in the SI LP and mLN, we analysed DC isolated from SI LP and mLN for expression of TIM-4 at different time points after CT treatment. CD103+ CD11b+ TIM-4+ DC were first detected in the SI LP 8 hours following CT treatment, peaked in frequency at 17 hours, and steadily declined thereafter. Interestingly, a fraction of MHCII^hi^ CD103+ CD11b+ DC in the mLN already express TIM-4 at steady state; however, their TIM-4 expression levels and frequency significantly increased by 12 hours after CT treatment, a short time after they first appeared in the LP (Fig 4E). Together these data suggest that the CD103+ CD11b+ TIM-4+ DC found in the mLN after CT treatment are a recent migrant population from the SI LP.

### TIM-4 expression is associated with increased uptake of apoptotic bodies and antigen by cultured intestinal APC

**Fig 5:**
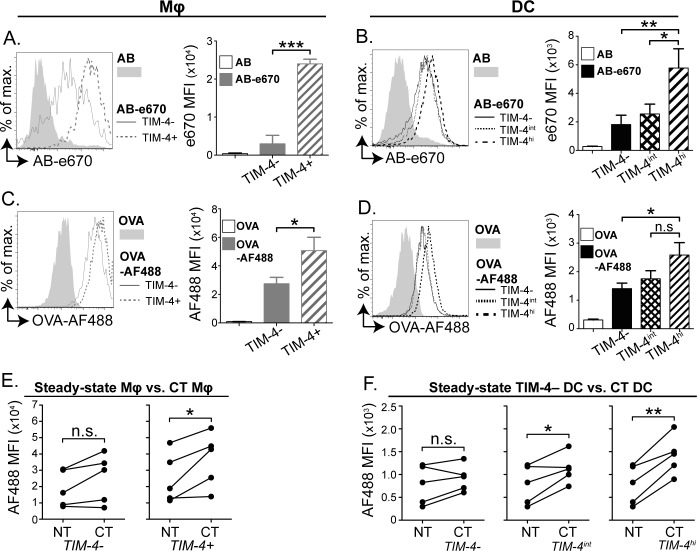
TIM-4 expression is associated with enhanced endocytic ability in APC from the SI. Cells were isolated from the SI LP of CT treated mice and enriched for CD11c+ cells by magnetic selection. (A) CD11c+ cells were co-cultured with e670 labelled (AB-e670) or unlabelled apoptotic bodies (AB) for 1 hour and TIM-4+ and TIM-4− macrophages assessed for e670 fluorescence by flow cytometry. (B) As in A, except TIM-4^hi^, TIM-4^int^ and TIM-4− DC were assessed for e670 fluorescence. (C) CD11c+ cells were co-cultured with ovalbumin (OVA)-AF488 or OVA for 1 hour and macrophages assessed for AF488 fluorescence by flow cytometry. (D) As in C, except TIM-4^hi^, TIM-4^int^ and TIM-4− DC were assessed for AF488 fluorescence. Histograms show data representative of 3–5 independent experiments and bar graphs show median fluorescent intensity (MFI)±SEM of data pooled from 3–5 independent experiments. Statistical significance was determined by one-way ANOVA with Bonferroni post-test. n.s. p >0.05, * p <0.05, ** 0.001<p<0.05, *** 0.0001<p<0.001. (E) As in C, except AF488 fluorescence was compared between macrophages populations isolated from CT and untreated (NT) mice. (F) As in D, except AF488 fluorescence was compared between DC populations isolated from CT and TIM-4– DC from untreated (NT) mice. Graphs show median fluorescent intensity (MFI)±SEM of data pooled from 5 independent experiments. Statistical significance was determined by two-way ANOVA. n.s. p >0.05, * p <0.05, ** 0.001<p<0.05.

As a receptor for PtdSer, TIM-4 has been described to play a role in the recognition and uptake of apoptotic cells by macrophages [29, 36]. However, TIM-4 associated apoptotic cell uptake has not been investigated in the SI or among DC. To address this, total CD11c+ cells were enriched from the SI of CT-treated mice by magnetic bead sorting and cultured with apoptotic thymocytes labelled with the fluorescent dye e670. After 1 hour of co-culture, DC and macrophage populations were identified by flow cytometry and assessed for e670 fluorescence. TIM-4+ macrophages had a significantly higher e670 fluorescent signal compared to their TIM-4− counterparts (Fig 5A). Furthermore, among DC, uptake correlated with the level of TIM-4 expression, with TIM-4^hi^ DC acquiring more apoptotic material than TIM-4^int^ and TIM-4− DC (Fig 5B). Importantly, the detected e670 signal was indicative of apoptotic cell uptake rather than cell surface association as DC from cultures maintained at 4°C had limited e670 signal (S4A Fig).

Further experiments examined the uptake of fluorescent ovalbumin (OVA-AF488) by the same APC populations. Analysis by flow cytometry showed significantly higher levels of OVA-AF488 uptake by TIM-4+ compared to TIM-4− macrophages (Fig 5C). Similarly, TIM-4^hi^ DC acquired significantly more antigen than TIM-4− DC. Although not statistically significant, TIM-4^hi^ DC consistently acquired more antigen than TIM-4^int^ DC suggesting an association between the level of TIM-4 expression and antigen uptake ability (Fig 5D). Given this and the association of TIM-4 with the activation status of SI APC, it was of interest to compare the antigen uptake potential of APC exposed to CT or in the steady-state. TIM-4− macrophages and DC from CT-treated mice trended to have higher OVA-AF488 fluorescence compared to cells from untreated controls; however, this trend was not statistically significant. Conversely, TIM-4+ macrophages from CT-treated mice acquired significantly more antigen compared to TIM-4+ macrophages from untreated mice. TIM-4^hi^ and TIM-4^int^ DC also acquired significantly more antigen than steady-state TIM-4− DC (Fig 5E and 5F). Again, cultures maintained at 4°C showed that this assay revealed uptake of OVA rather than binding of OVA to the cell surface (S4B Fig).

Together these data show that activated SI DC and macrophages are more efficient at taking up soluble antigen than steady-state APC. Furthermore, TIM-4 expressing APC are more efficient at antigen and apoptotic cell uptake compared to their TIM-4− counterparts. Overall, these observations suggest that the phenotypic changes to SI APC associated with CT exposure enhance their ability to acquire antigen.

## Discussion

The steady-state SI LP APC population is highly heterogeneous and complex, consisting of multiple CD11c+ macrophage and DC subsets that each have different propensities for antigen capture, migration and T cell priming. Currently, it is not clear how each LP APC population contributes to the initiation of intestinal effector immune responses. We show that following administration of the mucosal adjuvant CT, CD103+ CD11b+ LP DC selectively adopt an activated phenotype indicated by their increased expression of co-stimulatory molecules, and their increased frequency and number in the mLN. Furthermore, activated CD103+ CD11b+ DC acquired expression of the scavenger receptor TIM-4. Importantly, the amount of TIM-4 expressed by DC closely correlated with their activation state as well as their capacity for uptake of apoptotic cells and soluble antigen. Together these findings suggest that, in the presence of a mucosal adjuvant, CD103+ CD11b+ DC respond phenotypically and functionally to promote the priming of naïve T cells in the mLN.

In line with previous reports, we identified four LP DC subsets based on expression of CD103 and CD11b with CD103+ CD11b+ DC being the most abundant [12, 13, 21, 39]. At steady-state, LP DC subsets did not express TIM-4; however, following CT treatment, a proportion of CD103+ CD11b+ DC expressed TIM-4 which coincided with their up-regulation of co-stimulatory molecules. Importantly, TIM-4 expression and activation were restricted to this subset, and other LP DC subsets including the CD103- CD11b+ subset recently identified to be a CCR2+ CX3CR1^int^ population implicated in promoting effector T cell responses [19, 21] were unaffected following CT treatment. A previous study has shown that CD11c+ cells from the SI express increased TIM-4 message and protein following *in vivo* exposure to *Staphylococcus enterotoxin B* [26]. However, since the publication of that study, it has become apparent that intestinal CD11c+ cells comprise of both DC and macrophage populations, which makes it difficult to interpret those results [12, 13]. Other studies have shown that TIM-4 is expressed on lung draining LN DC in response to the TLR3 ligand PolyI:C [40] and on bone marrow-derived DC in response to *in vitro* exposure to LPS and CT [25, 27]. Together with our observations, the findings suggest that TIM-4 is not expressed on steady-state DC in tissues but is up-regulated during the process of activation in response to immunostimulatory agents including TLR ligands and bacterial toxins.

LP macrophages are typically classed as a homogenous population identified by their expression of CD64 and/or CX3CR1. However, data presented in this study suggests that the CD11c+ macrophage population may be heterogeneous as expression of TIM-4 defined two clear macrophage subsets. Microarray data has shown the presence of TIM-4 mRNA within flow sorted macrophage populations from the SI, spleen and peritoneum [40]. Our flow cytometry data clearly shows that cell surface expression of TIM-4 is restricted to 75% of the total SI macrophage population, which is consistent with observations made with peritoneal macrophages [31]. In addition, TIM-4+ SI macrophages up-regulated co-stimulatory molecules to a much greater extent than TIM-4− macrophages following oral administration of CT, suggesting that these macrophage subsets may differ in maturation stage, or their ability to recognise and respond to CT.

CT is known to exert its adjuvant effects by directly interacting with APC and inducing their maturation [41, 42]. This maturation is dependent on APC expression of cell surface GM1 ganglioside receptors and membrane-bound Gsα suggesting that CT requires GM1 to bind to APC and gain entry to the cell where it can modulate Gsα activation and ultimately APC maturation [41–43]. A recent study has shown that activation of CD11c+ DC preferentially occurs among the splenic CD11b+ population following intravenous delivery of CT, suggesting that CT has distinct effects on different DC subsets [43]. In this study, we show that CD103+ CD11b+ TIM-4+ intestinal DC selectively up-regulated co-stimulatory molecules and were found at a significantly higher frequency in the mLN following CT treatment. Interestingly, we consistently observed that only half of the total CD103+ CD11b+ population up-regulate CD86 and express TIM-4 in response to CT *in vivo*. Similarly, half of CD103+ CD11b+ DC activated *ex vivo* express TIM-4. This indicates that there may be heterogeneity within the CD103+ CD11b+ population, particularly in terms of ganglioside receptor expression. Furthermore, differences in the positioning of CD103+ CD11b+ DC within the LP may impact their accessibility to the intestinal lumen and direct interaction with CT. Together this data shows that a proportion of the CD103+ CD11b+ SI DC population, as identified by TIM-4 expression, selectively respond to CT by maturing and migrating to the mLN. As these processes are essential for the initiation of effector T cell responses this data suggests that CD103+ CD11b+ TIM-4+ DC are likely contributing to the adjuvant effect of CT in the intestine.

Despite the necessity for DC to transport antigen to the mLN for presentation, it remains unclear how DC initially acquire antigen in the LP. Orally administered fluorescent antigen is predominantly taken up by epithelial cells lining the intestine and sessile CX3CR1+ (CD64+) macrophages are rarely detected within migratory DC populations [18, 22, 44]. Therefore, it has been suggested that antigen can be transferred from non-migratory cell populations to LP DC and thus can be transported to the mLN. Studies have demonstrated that DC can acquire antigen from goblet cells and CX3CR1+ macrophages at steady-state but it is unclear how antigen acquisition occurs in the presence of microbial stimuli or adjuvants [18, 23]. On the basis of data shown here, we propose that DC may acquire antigen by taking up apoptotic vesicles derived from epithelial cells. Intestinal epithelial cells readily apoptose in response to pathogens, thus providing a pool of potential antigen for DC to sample. Consistent with this hypothesis, lung and LP DC have been shown to take up apoptotic material and present the apoptotic-associated antigens to T cells in the draining LN [45, 46]. Although presentation of antigens derived from apoptotic cells is associated with tolerance induction at steady-state [47, 48], the presence of infection or adjuvants may re-direct the response to active immunity [49].

In this study, we examined the uptake of apoptotic bodies by SI macrophages and found higher uptake by TIM-4+ macrophages compared to their TIM-4− counterparts. Similarly, when fluorescent OVA was added to *in vitro* LP APC cultures, TIM-4+ macrophages preferentially took up soluble antigen. Importantly, we found that activated CD103+ CD11b+ DC also expressing TIM-4 showed significantly greater uptake of soluble antigen and apoptotic cells *ex vivo* compared to TIM-4− DC. Interestingly, CD103+ CD11b+ DC expressing the highest level of TIM-4 and CD86 were better able to acquire apoptotic cells and OVA. Furthermore, macrophages and DC from CT-treated mice were more efficient at OVA uptake compared to their steady-state counterparts. These findings are in contrast to the view that activated DC are less efficient at antigen uptake compared to steady-state DC. However, others have suggested that while non-specific endocytosis is decreased among activated DC, receptor-mediated endocytosis through receptors such as DEC-205 is in fact increased [50]. TIM-4 is known to bind to PtdSer and TIM-4 expressing macrophages have been shown to assist with the recognition and uptake of apoptotic cells in the peritoneum and lung dLN [29, 31]. Thus, it is possible that TIM-4 may be directly involved in the increased antigen uptake observed. However, TIM-4− APC were also able to efficiently take up OVA and apoptotic bodies *in vitro*, suggesting that additional antigen uptake mechanisms besides TIM-4 are available in these cells. Therefore, we propose that cholera toxin activates SI DC to become better equipped to specifically acquire antigenic material from the environment, possibly through expression of molecules such as TIM-4.

In summary, we have shown that TIM-4 expression identifies a subpopulation of macrophages in the steady-state SI and is expressed on CD103+ CD11b+ DC upon activation following CT administration. Expression of TIM-4 was associated with an increased capacity for macrophages and DC to take up soluble antigen and apoptotic cells. Importantly, CD103+ CD11b+ TIM-4+ DC expressed high levels of co-stimulatory molecules and were detected in the mLN. Thus, CD103+ CD11b+ TIM-4+ DC are likely responsible for initiating effector immune responses in the presence of CT. Further study assessing the function and requirements for this SI DC population during immune activation will aid in the understanding of mechanisms by which SI APC orchestrate and regulate intestinal immunity.

## Supporting Information

S1 Fig CD26 and CD88 expression identify DC and macrophage populations in the SI LP, respectively. APC were isolated from the SI of untreated mice and analysed for expression of cell surface molecules by flow cytometry. (A) Dot plots show the gating strategy employed to identify distinct SI DC and macrophages (Mφ) populations. (B) Histograms compare expression profiles of cell surface molecules between DC and macrophage populations identified as in (A). (C) Dot plots show TIM-4 expression among CD103+ (black) and CD103- (grey) APC in relation to DC and Mφ specific markers. Data are from 1 of 3 independent experiments, each with 3 mice, that gave similar results.
(TIF)

S2 Fig The number of APC in the SI LP does not change following oral administration of mucosal adjuvant. APC were isolated from mice treated with CT 17 hours prior to analysis or untreated control mice (NT) and counted. (A) DC subset frequencies were identified by flow cytometry and were used to calculate cell numbers. (B) As in (A), except cell number was calculated for macrophage subsets. Bar graphs show mean±SEM for data compiled from 5 independent experiments.
(TIF)

S3 Fig Expression of co-stimulatory molecules on intestinal DC. (A) CD103+ CD11b− DC from untreated (NT) and CT treated mice were compared for expression of CD40 and CD80 by flow cytometry. Bar graphs show average median fluorescent intensity (MFI)±SEM. Statistical significance was measured by Student’s unpaired t-test. n.s. p >0.5, * p <0.05 (B) As in (A), except CD103− DC were assessed for CD40 and CD80 expression. (C) Cells were isolated from the SI of untreated mice and cultured for 4 hours in complete IMDM (cIMDM) with 500ng/mL lipopolysaccharide (from *Escherichia coli*, serotype 0111:B4) or 5μg/mL CT. CD86 and TIM-4 expression on CD103+ CD11b+ DC was assessed by flow cytometry. (D) DC were isolated from the SI LP of untreated mice (NT) and mice treated with CT at different time points and analysed for CD86 and TIM-4 expression by flow cytometry.
(TIF)

S4 Fig CD103+ TIM-4+ DC internalise apoptotic material and OVA antigen. Cells were isolated from the SI LP of CT treated mice and enriched for CD11c+ cells by magnetic selection. (A) CD103+ TIM-4^hi^ DC were assessed for e670 fluorescence following co-culture with e670 labelled (AB-e670) or unlabelled apoptotic bodies (AB) for 1 hour at 37°C or 4°C. (B) CD103+ TIM-4^hi^ DC were assessed for AF488 fluorescence following co-culture with ovalbumin (OVA)-AF488 or OVA for 1 hour at 37°C or 4°C.
(TIF)
