# Supplementary figures and images for: Activation-Induced TIM-4 Expression Identifies Differential Responsiveness of Intestinal CD103+ CD11b+ Dendritic Cells to a Mucosal Adjuvant
Source: PLoS One. 2016 Jul 5;11(7):e0158775. doi: 10.1371/journal.pone.0158775 (PMC4933342; doi:10.1371/journal.pone.0158775)

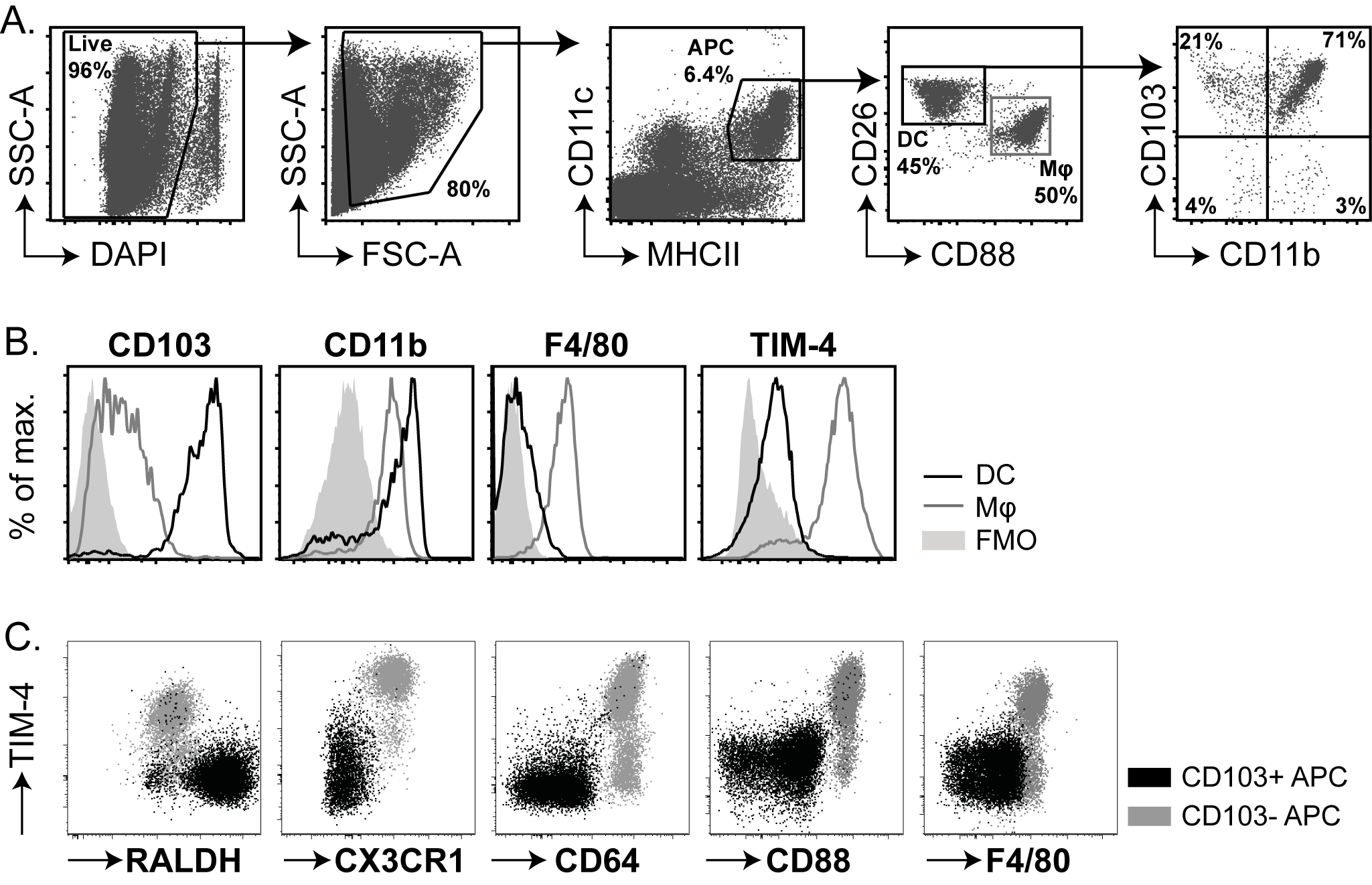

Supplement: S1 Fig — APC were isolated from the SI of untreated mice and analysed for expression of cell surface molecules by flow cytometry. (A) Dot plots show the gating strategy employed to identify distinct SI DC and macrophages (Mφ) populations. (B) Histograms compare expression profiles of cell surface molecules between DC and macrophage populations identified as in (A). (C) Dot plots show TIM-4 expression among CD103+ (black) and CD103- (grey) APC in relation to DC and Mφ specific markers. Data are from 1 of 3 independent experiments, each with 3 mice, that gave similar results. (TIF) [file pone.0158775.s001.tif]

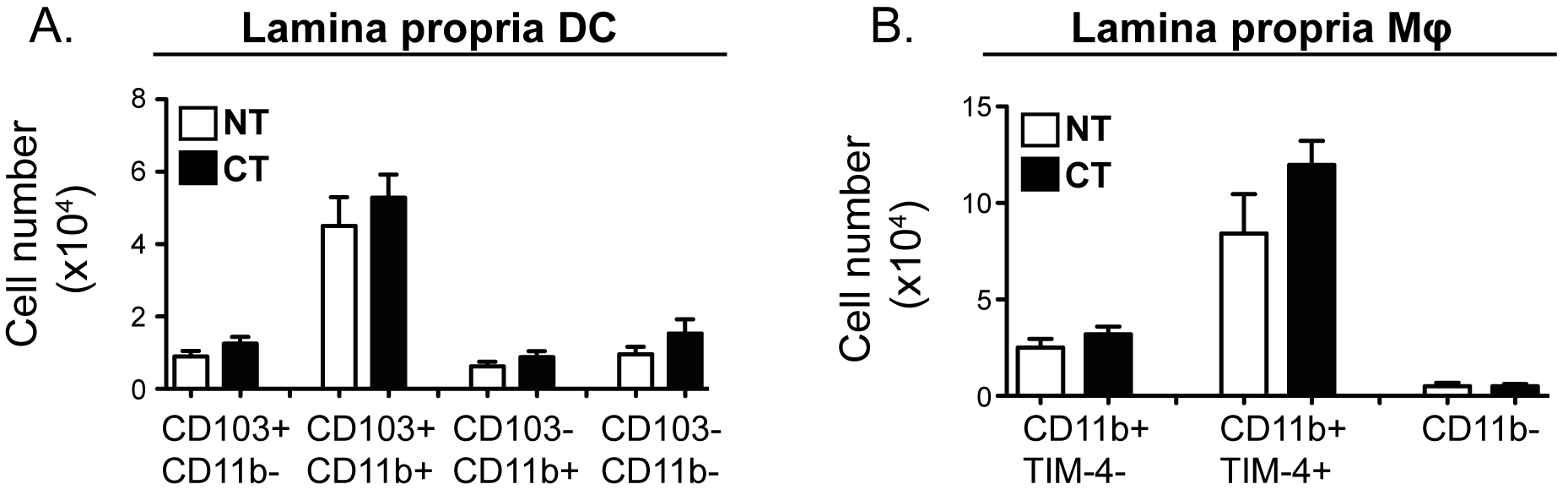

Supplement: S2 Fig — APC were isolated from mice treated with CT 17 hours prior to analysis or untreated control mice (NT) and counted. (A) DC subset frequencies were identified by flow cytometry and were used to calculate cell numbers. (B) As in (A), except cell number was calculated for macrophage subsets. Bar graphs show mean±SEM for data compiled from 5 independent experiments. (TIF) [file pone.0158775.s002.tif]

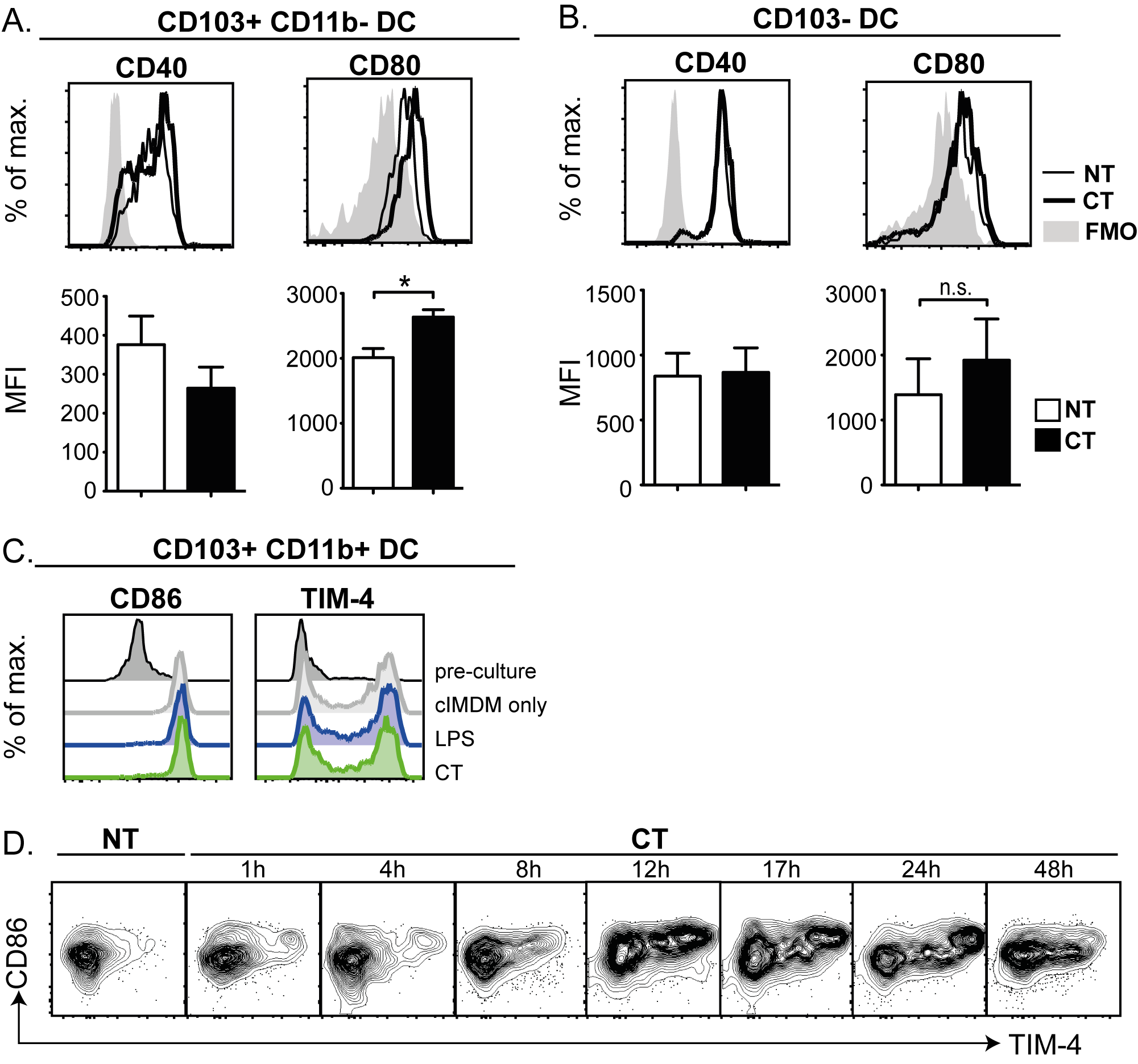

Supplement: S3 Fig — (A) CD103+ CD11b− DC from untreated (NT) and CT treated mice were compared for expression of CD40 and CD80 by flow cytometry. Bar graphs show average median fluorescent intensity (MFI)±SEM. Statistical significance was measured by Student’s unpaired t-test. n.s. p >0.5, * p <0.05 (B) As in (A), except CD103− DC were assessed for CD40 and CD80 expression. (C) Cells were isolated from the SI of untreated mice and cultured for 4 hours in complete IMDM (cIMDM) with 500ng/mL lipopolysaccharide (from Escherichia coli, serotype 0111:B4) or 5μg/mL CT. CD86 and TIM-4 expression on CD103+ CD11b+ DC was assessed by flow cytometry. (D) DC were isolated from the SI LP of untreated mice (NT) and mice treated with CT at different time points and analysed for CD86 and TIM-4 expression by flow cytometry. (TIF) [file pone.0158775.s003.tif]

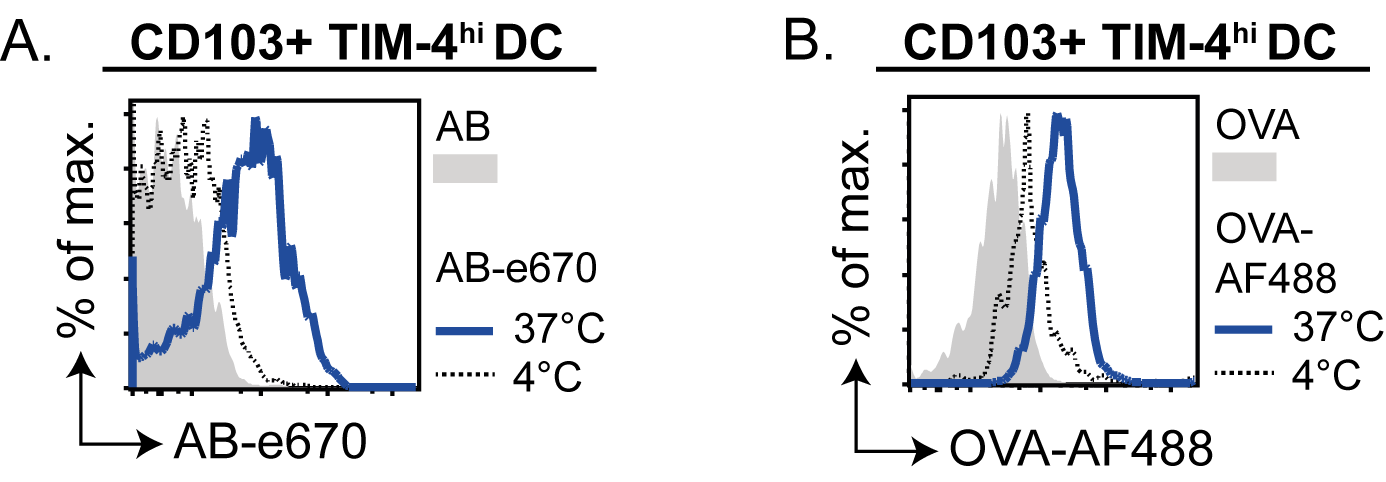

Supplement: S4 Fig — Cells were isolated from the SI LP of CT treated mice and enriched for CD11c+ cells by magnetic selection. (A) CD103+ TIM-4hi DC were assessed for e670 fluorescence following co-culture with e670 labelled (AB-e670) or unlabelled apoptotic bodies (AB) for 1 hour at 37°C or 4°C. (B) CD103+ TIM-4hi DC were assessed for AF488 fluorescence following co-culture with ovalbumin (OVA)-AF488 or OVA for 1 hour at 37°C or 4°C. (TIF) [file pone.0158775.s004.tif]
